# Early COVID-19 Government Communication Is Associated With Reduced Interest in the QAnon Conspiracy Theory

**DOI:** 10.3389/fpsyg.2021.681975

**Published:** 2021-08-31

**Authors:** Ho Fai Chan, Stephanie M. Rizio, Ahmed Skali, Benno Torgler

**Affiliations:** ^1^Queensland University of Technology, Brisbane, QLD, Australia; ^2^Centre for Behavioural Economics, Society and Technology (BEST), Brisbane, QLD, Australia; ^3^Department of Marketing, University of Groningen, Groningen, Netherlands; ^4^Melbourne School of Psychological Sciences, University of Melbourne, Melbourne, VIC, Australia; ^5^Department of Global Economics and Management, University of Groningen, Groningen, Netherlands; ^6^Center for Research in Economics, Management, and the Arts (CREMA), Zürich, Switzerland

**Keywords:** conspiracy theories, QAnon, COVID-19, coronavirus, government risk communication, blame allocation

## Abstract

Does inadequate risk communication during uncertain times trigger the rise of conspiratorial ideas? We hypothesize that, where government COVID-19 risk communication started early, as measured by the number of days between the start of the communication campaign and the first case in the country, citizens are less likely to turn to conspiratorial explanations for the pandemic, which typically assign blame to powerful actors with secret interests. In Study 1a, we find strong support for our hypothesis in a global sample of 111 countries, using daily Google search volumes for QAnon as a measure of interest in QAnon, which is a conspiracy theory contending, among other things, that COVID-19 is a conspiracy orchestrated by powerful actors and aimed at repressing civil liberties. The effect is robust to a variety of sensitivity checks. In Study 1b, we show that the effect is not explainable by pre-pandemic cross-country differences in QAnon interest, nor by ‘secular’ rising interest in QAnon amid the pandemic. A one-standard deviation (26.2days) increase in communication lateness is associated with a 26% increase in QAnon interest. In pre-registered Study 2, we find limited support for the proposition that early communication reduces self-reported pandemic-related conspiratorial ideation in a sample of respondents from 51 countries. Overall, our results provide evidence that interest in extreme ideas, like QAnon, are highly responsive to government risk communication, while less extreme forms of conspiracism are perhaps less so.

## Introduction

The rapid spread of the highly contagious and deadly COVID-19 virus, since its emergence in December 2019, has led to a global pandemic—a state of affairs not seen since the 1918 Spanish Flu (see Ashton, 2020 for a comparison).^1^ Governments around the world communicated with the public about the virus with varying degrees of swiftness: the data from Hale et al. (2021), which we delve into in Study 1, show that there is substantial heterogeneity in how quickly governments began communicating with the public about COVID-19. In this paper, we ask whether the swiftness of government risk communication can explain the spread of COVID-19-related conspiracy theories, which typically seek to assign blame for the pandemic to powerful actors with secret agendas.

Our main hypothesis is that, where government risk communication is slow, there are opportunities for people to ‘fill in the blanks’ with conspiratorial ideas which attempt to rationalize the situation at hand, namely, the pandemic. The COVID-19 pandemic is an ideal breeding ground for the spread of false narratives: a sudden environment of extreme angst, frustration, and fear materialized, which in the minds of many people could not have been foreseen, and thus requires an extraordinary explanation. As a matter of fact, apart from the virus itself, a hallmark feature of the COVID-19 pandemic has been the proliferation of conspiracy theories on social media, a pattern which began early on during the pandemic (Van Bavel et al., 2020a). More generally, as shown by the folklorist Jon D. Lee (2014) in his book *An Epidemic of Rumors*, pandemics and epidemics, from AIDS to H1N1 and SARS, commonly give rise to rumours and conspiratorial narratives. Thus, we hypothesize that false narratives spread where governments do not communicate swiftly with the public about the virus.

We test our hypothesis using Google search data as a proxy for interest in the QAnon conspiracy theory in Study 1 and find strong support for our hypothesis. Our motivation for studying QAnon is that it is an integral part of what Rosenblum and Muirhead (2020, p. 35) define as the ‘new conspiracism’, which is an ‘active assault on democracy’. QAnon is an extreme conspiratorial movement which blames a supposed secret cabal of left-leaning politicians for many real or perceived ills, including the pandemic. QAnon’s spread is of current policy concern, as the group has been designated a terror threat by the FBI as early as 2019.^2^ Central to QAnon lore is the dangerous belief that the pandemic is a hoax,^3^ which makes QAnon a phenomenon deserving of empirical investigation. Importantly, the human cost of becoming embroiled in QAnon is also staggering, as evidenced by the stories of individuals ‘losing’ loved ones to the cult-like nature of QAnon,^4^ which motivates us to study QAnon in Study 1. We also test our hypothesis using self-reported conspiratorial beliefs in a sample of approximately 40,000 respondents from 51 countries from the International Collaboration on Moral and Social Psychology (Van Bavel et al., 2020b), in the pre-registered Study 2. Our hypothesis finds limited support in Study 2, which suggests that not all conspiratorial ideas respond equally largely to government (in)action. Our results provide ample caution about the responsiveness of interest in extreme ideas, such as QAnon, to government risk communication.

Our work contributes to a well-established area of investigation in psychology and across the social sciences, which is the study of conspiracy theories (for overviews, see Lewandowsky and Cook, 2020; van der Linden et al., 2021) and of false beliefs more generally (O’Connor and Weatherall, 2019). The phenomena of scapegoating and conspiracy theories ensuing from pandemics have a long history, dating back at least to the plague of Cyprian in Roman times (Retief and Cilliers, 2000). Conspiracy theories and false narratives, more generally, tend to circulate more in times of uncertainty or complexity as a way of trying to make sense of what is going on in the world around us. These usually relate to clandestine government plans, elaborate murder plots, or paranoia about powerful groups, thinking they are sinister or have ‘hidden agendas’, and persist even when there is no decisive evidence for them (Lewandowsky and Cook, 2020). People ‘fill in the gaps’ with their own explanations as a way of relieving feeling of anxiety and stress (Douglas et al., 2017)—even going as far as assigning blame or responsibility to certain individuals or groups to fulfil their epistemic need for an explanation, with the scapegoating of Jews during the Black Death being a salient example. We thus contribute to a nascent literature analysing interest and beliefs in conspiracy theories in relation to the COVID-19 pandemic (see, for example, Cassese et al., 2020; Enders et al., 2020; Imhoff and Lamberty, 2020; Miller, 2020; Sternisko et al., 2020; Stoica and Umbreș, 2020; Uscinski et al., 2020; Chan et al., 2021; Šrol et al., 2021).

Our work also contributes to a strand of research in the crisis and risk communication literature, which emphasizes the benefits of communicating early (see, e.g., Heath and O’Hair, 2009; Coombs and Holladay, 2011). In mock criminal trials, Dolnik et al. (2003) show that revealing damaging information about oneself (a strategy known as ‘stealing thunder’) without waiting for others to reveal it first is beneficial to the party revealing the information. In an organizational context, Arpan and Roskos-Ewoldsen (2005) show that stealing thunder results in higher credibility ratings for the disclosing organization. Williams and Treadaway (1992) argue that the Exxon corporation’s slow communication response to the grounding of the Exxon Valdez oil tanker in Alaska played a driving role in the failure of Exxon’s communication strategy. In the context of health communication, Covello (2003, p. 5) specifically defines as best practice to ‘demonstrate respect for persons affected by risk management decisions by involving them early, before important decisions are made’. Thus, in the case of the COVID-19 virus outbreak, our findings complement the extant risk communication literature by showing that early communication about the virus has a chilling effect on the diffusion of conspiratorial narratives people turn towards, to ease their feelings from the uncertainty of the virus’ nature and spread. To the best of our knowledge, this paper is the first to quantitatively explore crisis communication during COVID-19 (see Malecki et al., 2021 for a discussion).

## Study 1A

### Data

#### QAnon

The origins of QAnon can be traced back to 28 October 2017, when a user of the internet forum 4chan began claiming that he or she was a high-ranking political insider working to inform the public about Donald Trump’s battle against a so-called criminal deep state (Gallagher et al., 2020, p. 3). The username of the person posting this claim was Q, which is the highest level of security clearance in the United States, thus appearing to corroborate Q’s claim that they are a high-ranking insider.^5^ Q’s identity remains unknown, and it is unclear whether multiple people have posted on 4chan while claiming to be Q. As Gallagher et al. (2020, p. 3) put it, ‘The QAnon theory now connects antivaccine, anti-5G conspiracies, antisemitic and antimigrant tropes, and several bizarre theories that the world is in the thrall of a group of paedophile elites set on global domination in part aided by ritualistic child sacrifice’. As disjointed as QAnon might sound, there is no doubt it has captured the attention of many around the world and is far from limited to the United States (Gallagher et al., 2020), where it has been designated a domestic terror threat.

We use daily country-level Google search volumes to measure interest in QAnon from 1 January to 24 May 2020. We use the latter as our cut-off date because it is the day before George Floyd was murdered by Minneapolis police. Floyd’s murder gave rise to large popular protests, leading at least some QAnon followers to conclude that the protests were staged by a ‘deep state’ to harm Donald Trump’s re-election chances (Gallagher et al., 2020).

Using Google searches as a proxy for interest in QAnon follows in the footsteps of Stephens-Davidowitz (2014), who shows that racial animus, as proxied by search terms for the n-word, cost Barack Obama about 4 percentage points of the national popular vote. While we cannot know for certain that searches for QAnon reflect belief in QAnon, Madestam et al. (2013) provide evidence that Google searches are correlated with actual political behaviour. They document rising interest in the Tea Party between 2009 and 2011, as measured by Google searches, which accompanied increased attendance at Tea Party rallies. In Supplementary Figure S1, we also provide evidence that Google searches for Jo Jorgensen, the Libertarian Party candidate to the US presidency, predict votes for Jo Jorgensen at the state level, such that Google searches are indicative of political behaviour.^6^ A major advantage of using Google searches as a proxy for interest in QAnon is that Google searches do not suffer from social desirability bias (Stephens-Davidowitz, 2014). This is particularly true for sensitive questions, as is the case for conspiratorial ideas.

Google search volumes for a given topic are measured as a share of all Google searches for a given country and date, and range from 0 (date with the least interest) to 100 (date with the most interest). For example, Google searches for the weather in the United States (Supplementary Figure S2) are approximately constant for the first 2months of 2021 and peak markedly on February 15, which was around the start of winter storm Viola.^7^ Because Google search volumes for QAnon are *relative* to other searches, higher numbers do not mean that people are at home because of the pandemic searching for more of everything. Instead, higher searches for QAnon specifically mean that searches for QAnon are becoming more frequent relative to all other searches. Since each country has data ranging from 0 to 100, and we are interested in cross-country comparisons, we adjust the original data to reflect cross-sectional differences in search volumes between countries. We adjust by using cross-sectional search intensity from Google trends, which ranks countries from most searches (100) to least (0), for a given time period. Austria is the country which sees the most searches for QAnon and receives a score of 100. We thus leave Austria’s time-series data unchanged. The United States has a cross-sectional score of 83, meaning that its searches for QAnon are 83% as large as Austria’s; we therefore multiply all daily search volumes for the United States by 0.83, in order to make them comparable with Austria’s. We perform this adjustment for all countries in the data set.

#### Late Campaign

For a given country, we measure the timeliness of government COVID-19 communication as the number of days between the date of the first case of COVID-19 in the country and the date on which government officials began communicating with the public about COVID-19. Both of these variables are drawn from the Oxford COVID-19 Government Response Tracker (OxCGRT; Hale et al., 2021) data set, the main source of information on governmental responses to the pandemic, from which we also draw several control variables as detailed below. The OxCGRT data set records the first case of COVID-19 in New Zealand on 28 February 2020; the earliest government communication began on 22 January 2020, thus giving New Zealand a value of value for *Late Campaign=*−37, as their government began communicating 37days before the first case. Alternatively, we also define another version of *Late Campaign* relative to the first death in the country, rather than relative to the first case. Supplementary Tables S1 and S2 in the Supplementary Material provide descriptive statistics and definitions and sources, respectively, for all variables used in this paper.

#### Sample Composition and Country-Level Descriptive Statistics

Our main two variables described above are available for 111 countries and territories. The full list of countries included in either Study 1 or 2 is provided in Supplementary Table S3, along with country-level summary statistics for key variables of interest.

### Methods

We estimate the following regression model:

$$\begin{array}{l} QAnon_{it}=\alpha_{0}+\alpha_{1}LateCampaign_{i}+\alpha_{2}QAnon_{i,t-1}\\ +X_{it}\gamma +\epsilon_{it} \end{array}$$ (1)

where the dependent variable, *QAnon*, measures the volume of Google searches for the QAnon topic of QAnon in country *i* on day *t*, α_0_ is a constant term, *Late Campaign* is the number of days elapsed between the start date of government COVID-19 communication campaigns and the first case of the virus (or first death from the virus) in the country, **X** is a vector of country-level control variables, and ε is an error term. Larger values of *Late Campaign* denote a later campaign, which we hypothesize to lead to larger interest in QAnon. Because search volumes for QAnon tend to be correlated from one day to the next in a given country, we control for the first lag of the dependent variable in all regressions. This is a conservative choice, since the coefficient of *Late Campaign* will reflect differences in search volumes between countries that cannot be explained by past search volumes. Our standard errors are heteroskedasticity and autocorrelation consistent, and are clustered over countries. Our results are robust to alternate estimation methods and clustering strategies (Supplementary Figure S3).

### Results

**Figure 1:**
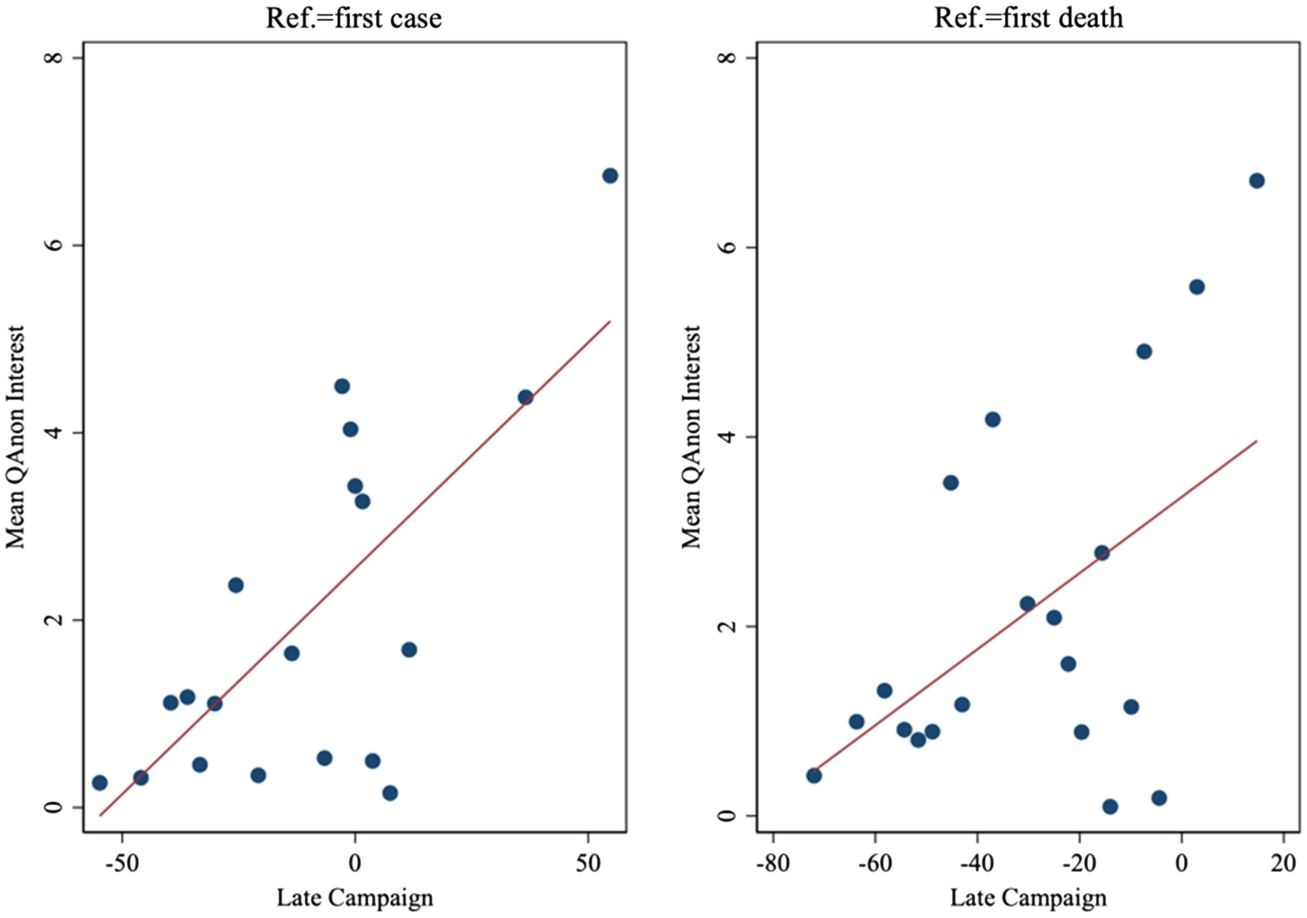
Binned scatterplot of interest in QAnon and *Late Campaign*.

Figure 1 displays a binned scatterplot of the basic relationship in the data. The mean interest in QAnon increases as *Late Campaign* increases; this is true both when *Late Campaign* is defined relative to the first case of COVID-19 in the country (left-hand side panel) or when it is defined relative to the first death (right-hand side panel).

Table 1 presents the main regression results. The top panel of the table, Panel A, presents results using the first case of COVID-19 as a reference point against which government communication campaign starts are measured, while Panel B uses the first death as the reference point. Model 1 presents the baseline estimates: a one-day increase in communication lateness is associated with a statistically significant 0.008–0.01 increase in searches for QAnon. The mean of the dependent variable is 2.05 approximately; the estimated effect therefore represents an increase in the order of 0.4–0.5% from the mean. Another quantity of interest is the effect of a one-standard deviation (26.2days) increase in communication lateness, which is associated with a 11–13% increase in searches for QAnon. The effects we estimate are therefore sizable. Since our models treat the first lag of *QAnon* as an exogenous variable, our estimates are also conservative and should be understood as a lower bound.

**Table 1:** Main results from Study 1.

	(1)	(2)	(3)	(4)
**A. *Late Campaign* relative to first COVID-19 case in country**				
Late Campaign	0.0102***	0.0129***	0.0105***	0.0134***
	[0.0037]	[0.0041]	[0.0039]	[0.0043]
Continent FE		Yes		Yes
Day FE			Yes	Yes
Observations	15,969	15,969	15,969	15,969
Within R2	0.352	0.352	0.368	0.369
Overall R2	0.644	0.647	0.650	0.654
**B. *Late Campaign* relative to first COVID-19 death in country**				
Late Campaign	0.0083**	0.0101**	0.0085**	0.0105**
	[0.0038]	[0.0041]	[0.0039]	[0.0042]
Continent FE		Yes		Yes
Day FE			Yes	Yes
Observations	15,393	15,393	15,393	15,393
Within R2	0.354	0.354	0.371	0.371
Overall R2	0.644	0.647	0.651	0.654

Dependent variable=Google search queries for the QAnon topic. Late Campaign is the number of days between the start of government COVID-19 communication campaigns and the first COVID-19 case (Panel A) or death (Panel B) in the country. All specifications include a constant term. Standard errors in brackets are clustered over countries and are consistent for autocorrelation and heteroskedasticity. ^***^, ^**^ and ^*^ denote significance at the 1, 5 and 10% levels, respectively.

Model 2 builds up from Model 1, with the added inclusion of a vector of continent fixed effects, which play a crucial role in this setting. Continent dummies allow us to rule out the possibility that the results are driven by varying propensities to search for QAnon across geographic regions. It is entirely plausible that European Google users may have googled QAnon more than Asian users; if European countries also tend to have later communication campaigns, then our results from Model 1 would be confounded in the absence of continent dummies. Our estimates from Model 2 survive the inclusion of continent dummies, and if anything, increase slightly in size. Importantly, the coefficient from Model 2 has a within-continent interpretation: we find that, when comparing two countries within the same continent, the country with the earlier government communication has significantly less search activity for QAnon.

Model 3 controls for a full vector of day dummies, which allows us to control for global fluctuations in searches for QAnon. For example, QAnon may have been featured in a prominent news story and thus searched for on some days more than others, owing to reasons orthogonal to government communication; day dummies allow us to rule out that such patterns could be driving our results. Importantly, the inclusion of day dummies will flexibly account for the rising global popularity of QAnon, since the coefficient of each day dummy is the difference in searches for QAnon between the relevant day and the baseline day. Thus, QAnon’s rising global popularity will be reflected in higher coefficients for later day dummies, without constraining the daily step change to be linear. In Model 4, we control for both day fixed effects and continent fixed effects; the results are unchanged. Model 4 is the most demanding specification and is therefore our starting point for other specifications, from this point forward.

### Sensitivity

#### Covariates

**Figure 2:**
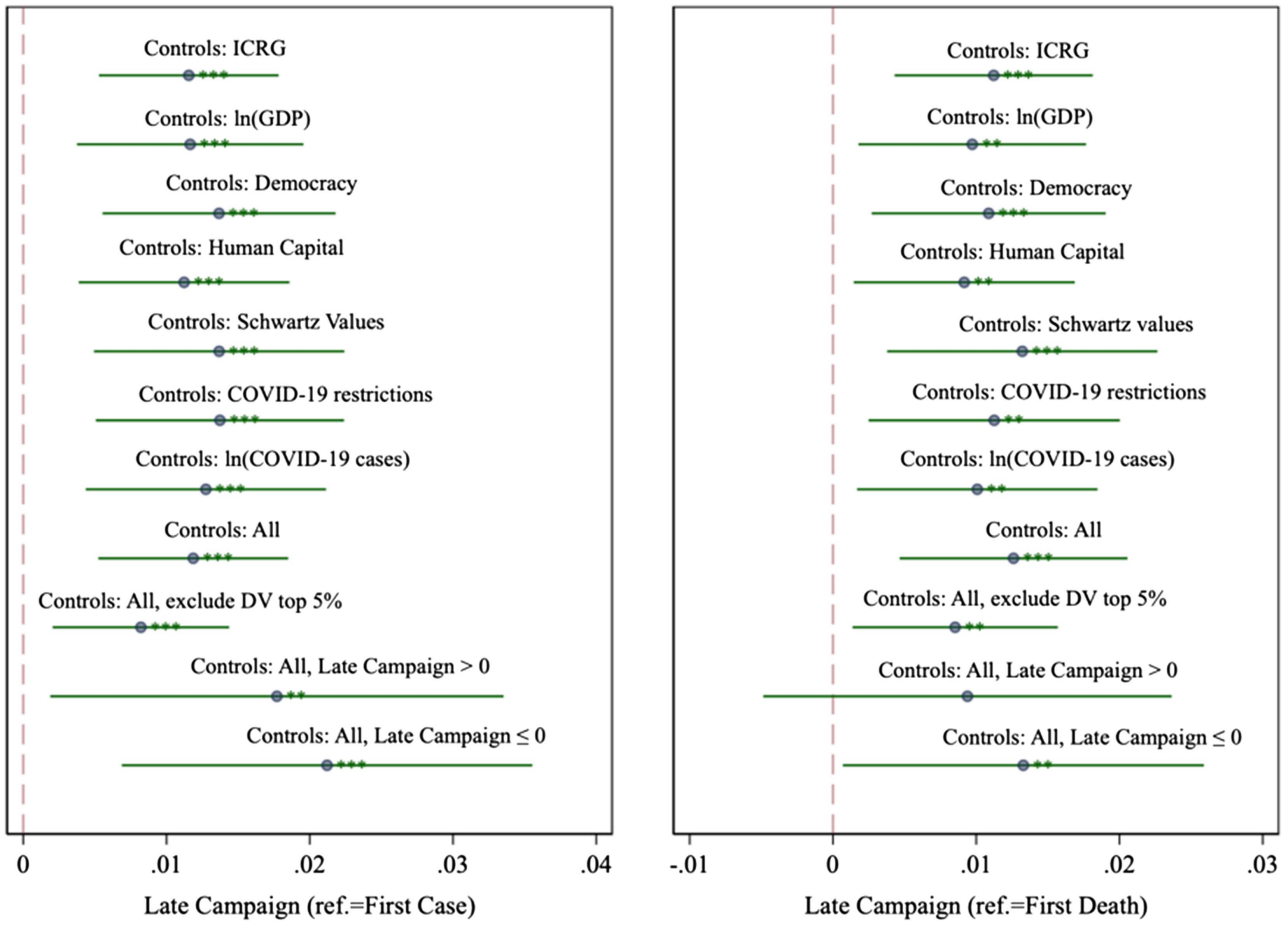
Sensitivity analysis. ***, ** and * denote significance at the 1, 5 and 10% levels. Significance levels are reported on the confidence interval spikes.

We consider an extensive set of factors which might correlate with both searches for QAnon and government’s ability or willingness to implement a quick communication campaign. In Figure 2, we report the coefficients of *Late Campaign* conditional on day fixed effects, continent fixed effects and seven sets of covariates. First, we control for 14 variables taken from the International Country Risk Guide (PRS, 2018), which capture the quality of the institutional environment. These variables are expert ratings on the quality of the local bureaucracy, corruption and government stability, among others (see Supplementary Table S2 for variable definitions). Second, we rule out that differences in economic development are driving the results, by controlling for the natural logarithm of *per capita* Gross Domestic Product. Third, we control for democracy, as measured in the Polity project (Marshall et al., 2013), which ranges from −10 (full autocracy) to +10 (full democracy). Fourth, we include an index of human capital from the Penn World Tables (Feenstra et al., 2015), since education might impinge on both search behaviour and government policy. Fifth, we control for differences in national culture using Schwartz’s (2006) seven cultural value orientations. Sixth, we control for an extensive set of COVID-19-related restrictions; seventh and finally, we account for the incidence of COVID-19 by controlling for the natural logarithm of *per capita* COVID-19 cases. Our estimates for *Late Campaign* remain large and statistically significant, and exhibit little variation in response to the inclusion of controls.^8^

#### Outliers

We check whether extreme values of *QAnon* are driving our results by excluding the observations with the 5% largest values of *QAnon*. The results, displayed in the third model from the bottom in Figure 2, show that *Late Campaign* decreases slightly in magnitude but remains significant. Wald tests for the equality of coefficients across models (dropping the top 5% of *QAnon* values *vs.* keeping all observations) fail to reject the null that coefficients are identical across models (*p*=0.07 and *p*=0.12, relative to first case or first death, respectively).

#### Early vs. Late Campaigns

It is possible that public opinion reacts differently to early messaging (*Late Campaign*≤0) than it does to late messaging (*Late Campaign*>0). In the former case, the government is communicating prior to the outbreak, with varying degrees of earliness; in the latter case, an outbreak has occurred, and the government is delaying communication. In the last two models of Figure 2, we disaggregate the analysis into those countries for which *Late Campaign*≤0 and those for which *Late Campaign*>0. Interestingly, in the right-hand side panel of Figure 2, where communication timeliness is measured relative to the first death, *Late Campaign* is insignificant when we restrict *Late Campaign* to be positive, but significant when we restrict it to be negative. This offers some indications that communicating well ahead of time (before the outbreak) may be the most effective strategy. We do interpret this difference with some caution, however, since Wald tests fail to reject the null of equality of the *Late Campaign* coefficients across the early-messaging and late-messaging periods (*p*=0.74 and *p*=0.42, relative to first case or first death of COVID-19 in the country, respectively).

#### Bottom-Censoring of the Dependent Variable

Google reports a search volume of 0 in a given time and place if the fraction of searches is below a certain threshold, such that the dependent variable is bottom-censored at 0. In Supplementary Figure S3, we report results using Tobit estimators, which take into account the censored nature of the dependent variable. Tobit coefficients of *Late Campaign* are larger than their OLS counterparts.

#### Alternate Error Structures

In Supplementary Figure S3, we report 95% confidence intervals for *Late Campaign* estimated with different clustering strategies, namely, clustering over days and double-clustering over countries and dates. The point estimates for *Late Campaign* remain statistically different from zero.

#### Placebo Analysis

If late-campaigning countries have some unobserved features that make them more likely to be high-QAnon-interest, it is possible that our results reflect the effect of some variable other than *Late Campaign*. To check whether our estimates may be affected by such unobserved factors that are correlated with *Late Campaign*, we conduct a placebo analysis. Specifically, we generate random values for *Late Campaign* and estimate their effect on QAnon, while conditioning on the full set of control variables. The rationale for the test is that, if *Late Campaign* is picking up the effect of another variable, then *Late Campaign* should perform no better than its placebo counterpart. We repeat the placebo-randomization 500 times, to obtain a distribution for the placebo *Late Campaign*. As Supplementary Figure S4 shows, the coefficient of actual *Late Campaign* lies beyond the 98.5th and 98th percentile of the placebo distributions, relative to the first case and first death, respectively. These estimates strongly suggest that *Late Campaign* predicts *QAnon* above and beyond the placebo, offering reassurance that our previous estimates are in fact detecting the effects of *Late Campaign* and not those of another variable.

#### Model Dependence

We consider whether our results are model-dependent by examining whether the patterns we document above are driven by idiosyncratic combinations of observations and control variables. Our starting point is the most demanding specification from Table 1 (Model 4), which includes day and continent fixed effects. For each variant of *Late Campaign* (defined relative to the earliest COVID-19 case or death), we run 500 iterations of our regression equation, including either (i) all control variables, and a randomly selected 50% of all observations, or (ii) all observations, and a randomly selected 50% of all control variables. We collect the resulting 2,000 test statistics for Late Campaign and plot them against their percentile rank in Figure 2. Overall, 92% of the t-statistics are above the rule of thumb critical value of 1.96 (shown by the dashed horizontal line), indicating that our results are not model-dependent.

## Study 1B

### Introduction

**Figure 3:**
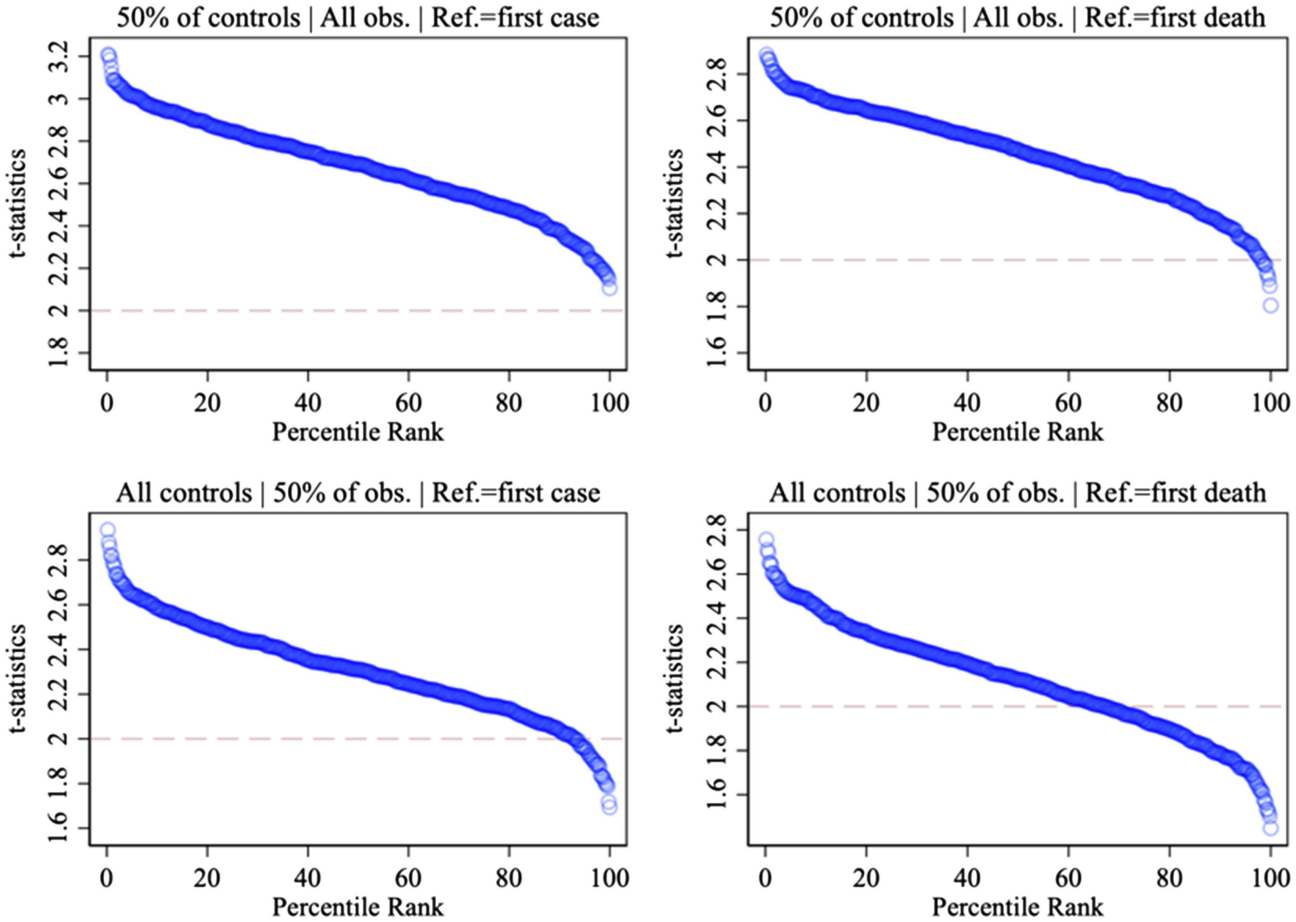
Model dependence: 2,000 test statistics and percentile ranks.

In Study 1a, we establish a correlation between government communication lateness and Google searches for QAnon. Our estimates suggest that a one-standard deviation increase in lateness is correlated with an approximately 12% increase in searches for QAnon. These results hold up to extensive scrutiny, as evidenced by Figures 2, 3. However, we cannot rule out that the observed pattern reflects pre-existing differences between countries. If late-communication countries had higher levels of QAnon searches prior to the pandemic, then it is possible that Study 1a is over-stating the importance of early communication.

### Methods

We estimate variants of Equation (2):

$$\begin{array}{l} QAnon_{it}=\beta_{0}+\beta_{1}Post_{it}+\beta_{2}LC_{i}+\beta_{3}\\ \left(Post_{it}*LC_{i}\right)+X_{it}\rho +\mu_{it} \end{array}$$ (2)

where *LC* is shorthand notation for *Late Campaign*, and *Post* is a dummy variable set equal to 1 from date *t*, for a given country, if either (i) government officials have started communicating about COVID-19, or (ii) the country has reported its first case of COVID-19. *Post* is thus equal to 1 from the day the virus is brought to the public’s attention, either *via* government communication or *via* the first local case. As such, *Post* accounts for differences in searches for QAnon across the pre- and during-pandemic periods, which allows us to rule out that any effect we see in Study 1a is driven by increased interest in QAnon due to the pandemic more generally, rather than to government communication timeliness. The coefficient of interest in Equation (2) is the coefficient of Post * LC, *ß_3_*, which captures differences in QAnon searches associated with communication timeliness in the post period, above and beyond: (i) secular trends captured by *Post* and, crucially (ii) pre-existing cross-country differences in QAnon searches that are associated with unobserved correlates of *Late Campaign*.

In Equation (2), the coefficient of *LC* is interpreted as the pre-pandemic correlation between QAnon searches and government communication. If countries with late government communication had higher QAnon searches to begin with, in the pre-pandemic period, then *LC* will account for those differences. The coefficient of Post * LC therefore informs us about the correlation between government communication and QAnon searches net of pre-existing differences and secular trends.

#### Main Results

**Figure 4:**
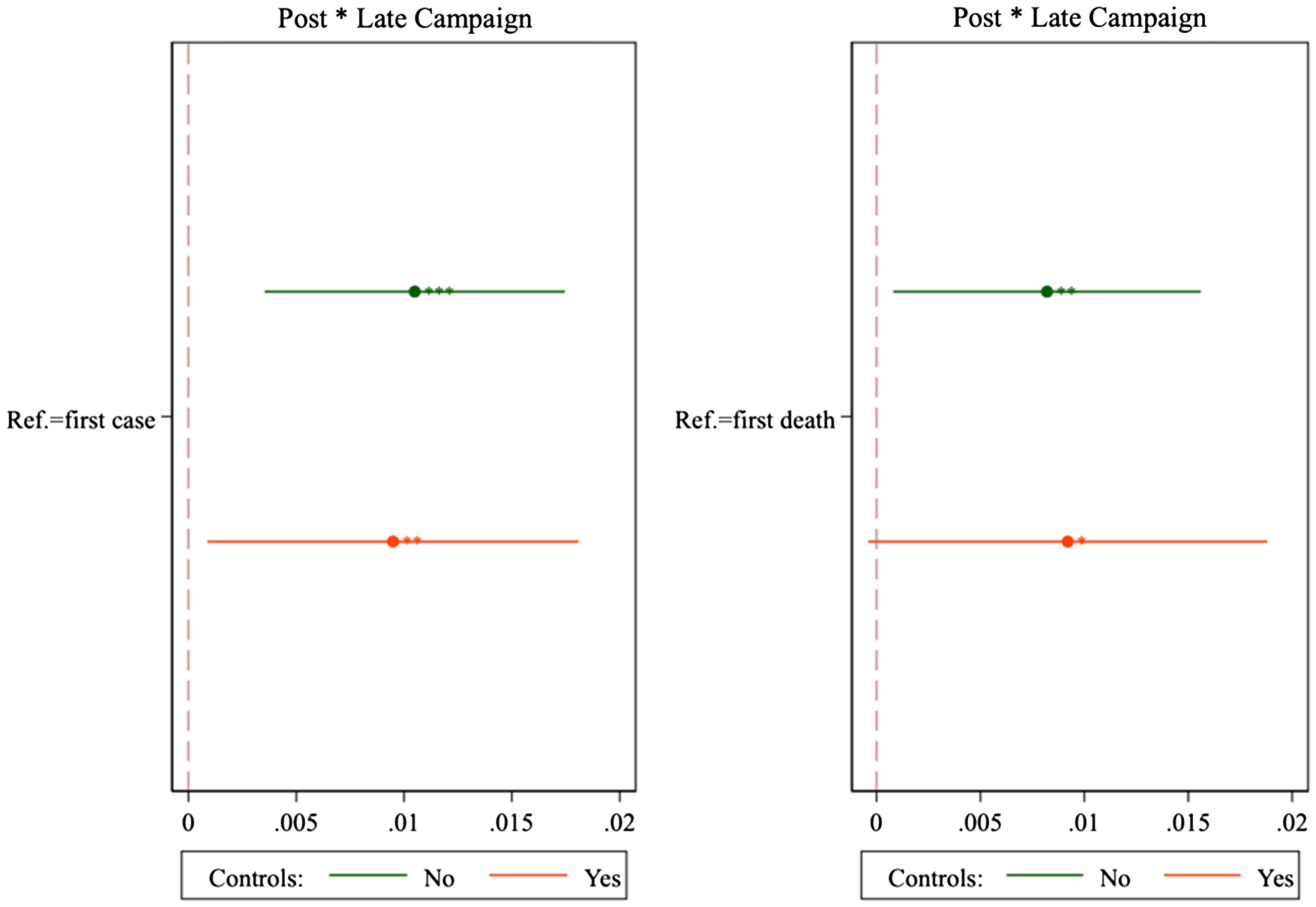
Coefficients and 95% CIs of Post * LC from Equation (2).

Figure 4 presents the results of estimating Equation (2) with either no covariates or the full set of covariates from Figure 2. The coefficient of Post * LC is large and significant throughout, indicating that our previous results were not driven by pre-existing differences in interest in QAnon or by increased interest in QAnon once the virus becomes known to the public. The mean of the dependent variable in the post period is approximately 2.6, and the coefficient of Post * LC is approximately 0.01. Thus, in the post period, having ruled out pre-existing differences in QAnon interest, the effect of a one-standard deviation (26.2days) increase in *Late Campaign* is a 26.2 * ((0.01+2.6) /2.6)=26% approximately increase in interest in QAnon, which is sizable.

### Regional Heterogeneity

**Figure 5:**
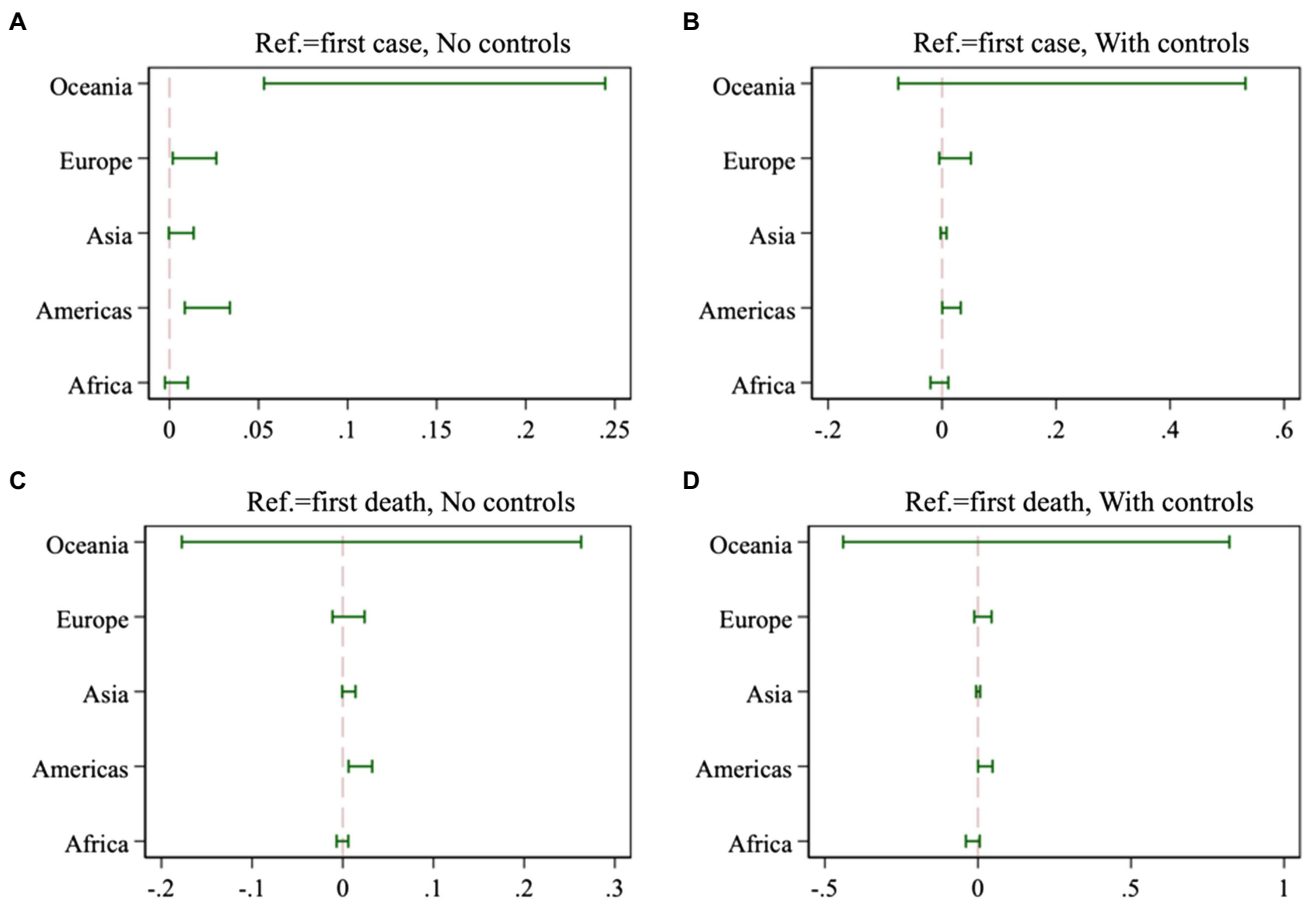
Coefficients of Post * LC from continent-specific regressions.

Does the pattern we document in the data differ across world regions? To explore this question, we estimate separate models for each continent and report the 95% confidence interval of Post * LC in Figure 5. The four panels of Figure 5 present results with and without control variables and separately for *LC* as defined relative to the first case of COVID-19 in the country (on the left-hand side) or relative to the first death (on the right-hand side). We find evidence of a heterogeneous relationship: while *Post* * *LC* is insignificant in Africa, the estimates are generally positive for other continents. In particular, the estimates are larger for the Americas, Oceania (which cannot be precisely estimated), and Europe, and positive but insignificant in Asia. One noteworthy limitation of this analysis is that the number of degrees of freedom is necessarily reduced when we split the sample across continents;^9^ still, it is interesting to note that there is some degree of regional heterogeneity at play.

## Study 2

### Background

Do the results presented in Study 1 apply exclusively to the QAnon conspiracy theory, or do they extend to other COVID-19-related conspiracy theories? To answer this question, we use data from the International Collaboration on the Social and Moral Psychology (ICSMP) of COVID-19 study (Van Bavel et al., 2020b). We pre-registered our analysis on the Open Science Framework at^10^, which was necessary in order to obtain the data.

### Materials and Methods

The ICSMP study surveyed 44,000 respondents from 67 countries on their attitudes and behavioural intentions related to the COVID-19 pandemic. The study is a large-scale collaboration involving research teams around the world; further details on the project, including a complete codebook, are available at^11^. Each team was asked to collect age and gender-representative data from their own country/territory. The original survey was created in English and translated as appropriate by local research teams, using the forward-backward translation method. Ethics approval was obtained from the University of Kent (approval ID number: 202015872211976468). The data we use in this study are well-balanced on sex (52.4% female) and smooth with respect to age, with no conspicuously missing age brackets (Supplementary Figure S5). A greater proportion of younger respondents is found in Africa and Asia, where fertility is relatively high. Shorter life expectancies in Africa are also visible in the data. Country-level descriptive information is available in the Supplementary of Van Bavel et al. (2020b).

The list of countries included in Study 3, along with the number of respondents per country, can be found in Supplementary Table S3. We follow our pre-analysis plan with two departures. First, in our pre-registration, we indicated that we would drop from the data set those respondents who gave the same number answer on two specified pairs of questions from the moral identity block of the survey, thus indicating that the respondent was not reading the question before answering. We also did not foresee that respondents could hold genuinely middle-of-the-road opinions, leading them to answer the pair of questions with 5 out of 10. This pattern is borne out in the data (Supplementary Figures S6, S7 and accompanying notes); we therefore keep those respondents who responded with 5 out of 10 on our flat-line detection questions, but exclude others as per our pre-registration. Second, following a recommendation from an anonymous reviewer, we exclude countries with fewer than 100 respondents. The results exactly following our pre-registration (exercising neither of the above departures) are shown in Supplementary Table S4.

Keeping in line with our pre-registration, we consider two dependent variables from the ICSMP, which we refer to as the ‘Authoritarian’ and ‘Financial’ conspiracy types in Table 2 below. *Authoritarian* is the degree of agreement, from 0 to 10, with the statement: ‘The coronavirus (COVID-19) is a conspiracy to take away citizen’s rights for good and establish an authoritarian government’. *Financial* is the degree of agreement with the statement ‘The coronavirus (COVID-19) is a hoax invented by interest groups for financial gains’.

**Table 2:** Main results from Study 2.

	(1)	(2)	(3)	(4)
	Authoritarian	Financial	Authoritarian	Financial
**A. *Late Campaign* relative to first COVID-19 case in country**				
Late Campaign	−0.0110	−0.0138*	0.0048	0.0135**
	[0.0080]	[0.0074]	[0.0055]	[0.0053]
Observations	39,069	39,066	38,528	38,525
R-squared	0.0073	0.0121	0.1361	0.1524
N. Countries	49	49	49	49
Continent FE			Yes	Yes
Country Controls			Yes	Yes
Demographics			Yes	Yes
**B. *Late Campaign* relative to first COVID-19 death in country**				
Late Campaign	−0.0075	−0.0076	−0.0019	0.0088
	[0.0085]	[0.0079]	[0.0060]	[0.0058]
Observations	39,069	39,066	38,528	38,525
R-squared	0.0026	0.0028	0.1360	0.1514
N. Countries	49	49	49	49
Continent FE			Yes	Yes
Country Controls			Yes	Yes
Demographics			Yes	Yes

Late Campaign is the number of days between the start of government COVID-19 communication campaigns and the first COVID-19 case (Panel A) or death (Panel B) in the country. FE=fixed effects. Demographics: age (continuous), gender (categorical) and marital status, has children (binary) and employment status (categorical). All specifications include a constant term. ^**^ and ^*^ denote significance at the 5, and 10% levels.

### Results

Table 2 presents the main results of Study 2. In Models 1 and 2, we estimate unconditional correlations between *Late Campaign* and either *Financial* or *Authoritarian*. The coefficients are statistically indistinguishable from zero, with the exception of Panel A Model 2, which is weakly significant (*p*=0.071). In Models 3 and 4, we control for differences between individual respondents as well as differences between countries by including continent fixed effects, the full set of country variables from Study 1, as well as respondent-level demographic^12^ variables (see Supplementary Table S2 and notes to Table 2 below). Once we rule out these confounders, there is tentative evidence that late communication provides a fertile breeding ground for conspiracism: the sign of *Late Campaign* is positive in three out of four cases and highly significant in one case (*p*=0.015, Panel A Model 4).

### Comparison of Results Across Studies

Is the pattern we observe in Study 2 idiosyncratic to the group of countries sampled in the ICSMP? In Supplementary Table S5, we report results from the full Study 1a specification for all countries which appear in both studies. The results show that the Study 1a patterns hold when the sample is restricted to be the same as in Study 2.

## Limitations

While Google searches (Study 1) present the clear advantages of being measured in near real-time, available across a wide range of geographies, and not subject to desirability biases, a noteworthy limitation of Google data is that they do not necessarily reflect being ‘taken in’ by the QAnon conspiracy theory. We do however have evidence from previous work (Madestam et al., 2013; Stephens-Davidowitz, 2014) and from Supplementary Figure S1 that Google searches correlate with actual political behaviour, such that our results are unlikely to reflect mere fleeting curiosity. Relatedly, it is also possible that the differences in the results across the two studies are driven by differences in measurement. A potential limitation is that the outcome variables used across studies are internet searches and self-reports, which present clear differences in measurement, and might thus hinder comparability across studies.

Another limitation of this paper is that we do not study other elements of government risk communication besides timeliness. One would expect that other facets of risk communication, including accuracy and consistency, also matter for the diffusion of false narratives: there are anecdotal reports, for example, of the public feeling misled by early calls for not using face masks (which were ostensibly directed at preventing mask hoarding) which were later reversed to recommended or even compulsory mask policies.^13^ We believe this is a fruitful area for future data collection efforts, as we know of no data set that takes stock of other facets of government risk communication beyond timeliness. Another potentially productive research agenda, going forward, would be to explore the dynamics of conspiratorial beliefs and government communication as contextual elements change. We leave these questions open for future research.

## Conclusion

In general, the capacity for belief is a core and dominant force in humans. As Fuentes (2019) points out, ‘[b]eliefs permeate our neurobiologies, bodies, ecologies and societies. They mediate the whole of human existence’ (p. 65). But beliefs are not always a good thing. There are also dangers inherent in such a capacity. Misleading beliefs about the world can threaten societies’ fabrics. In the long term, societal functioning depends on beliefs that are consistent with available evidence. However, an overload of information can result in a failure to properly process available information. As Herbert Simon (1983, p. 22) points out, we all ‘have modest computational abilities in comparison with the complexity of the entire world that surrounds’ us. Thus, it is worth exploring which mechanisms help society better respond, either to available evidence or to the spread of false beliefs. Investigating such mechanisms is particularly important in times of crises (such as pandemics) which can trigger prolonged uncertainty, feelings of fear and a sense of needing reassurance to cope with the challenging situation, often through assigning blame to others for the occurrence of the crisis. Although interest in conspiracy theories is different from actual belief, showing higher levels of interest can be a warning sign against exercising a sensible approach when drawing inferences from available facts.

In this paper, we have investigated the link between timely risk communication and the assignment of blame for the pandemic, as reflected by interest or belief in conspiratorial narratives. Our results indicate that a key mechanism in reducing the spread of interest in the QAnon conspiracy theory is the timely provision of risk communication regarding the emergency faced. In Study 1a, we showed that the earlier governments communicate about the virus, relative to the first instance of the virus in a given country, the lower the public’s interest in the destructive QAnon conspiracy theory – as measured by Google searches for QAnon in a sample of 111 countries and territories. In Study 1b, we showed that the results of Study 1a cannot be explained away by either of two crucial factors: (i) rising interest in QAnon in the COVID-19 era and (ii) pre-pandemic cross-country differences in interest in QAnon. Instead, interest in QAnon appears to rise specifically in response to late government risk communication about the virus, with a degree of regional heterogeneity, as effects were overall larger in the Americas, Oceania and Europe. These results should serve as a caution for policymakers in future developments with the COVID-19 pandemic and, in other crises, as they may arise: the late communication of risk can foster the rise of extreme ideas. We believe this is an important result, especially in a world where misinformation is rife.

In the pre-registered Study 2, we found only limited evidence of a relationship between government communication timeliness and self-reported beliefs in other conspiracies around the COVID-19 pandemic. Specifically, we did not find evidence that respondents in countries with later government communication think that COVID-19 is a conspiracy to establish an authoritarian government or a hoax perpetrated by interest groups for financial gains. Once we account for observable differences between countries and individual respondents, we do find evidence of higher conspiracy beliefs for the latter outcome, but not for the former. The overall picture emerging from Study 2 is thus mixed, with only limited indication that self-reported conspiracy beliefs respond to the timeliness of communication. Overall, we believe it is reassuring to observe that not all conspiratorial ideas respond equally to government (in)action.

## Data Availability Statement

The data analyzed in this study is subject to the following licenses/restrictions: Part of the data used for this article is proprietary (the International Country Risk Guide). We are happy to share our replication data with users who have access to the ICRG. Requests to access these datasets should be directed to PRS Group.

## Ethics Statement

Ethical review and approval was not required for the study on human participants in accordance with the local legislation and institutional requirements. Written informed consent for participation was not required for this study in accordance with the national legislation and the institutional requirements.

## Author Contributions

HC: conceptualization, formal analysis, and writing – review and editing. SR: conceptualization, writing – original draft, and writing – review and editing. AS: conceptualization, validation, formal analysis, and writing – original draft. BT: conceptualization, methodology, and writing – review and editing. All authors contributed to the article and approved the submitted version.

## Conflict of Interest

The authors declare that the research was conducted in the absence of any commercial or financial relationships that could be construed as a potential conflict of interest.

## Publisher’s Note

All claims expressed in this article are solely those of the authors and do not necessarily represent those of their affiliated organizations, or those of the publisher, the editors and the reviewers. Any product that may be evaluated in this article, or claim that may be made by its manufacturer, is not guaranteed or endorsed by the publisher.

## Footnotes

^1^For a historical overview on pandemics and their societal relevance, see Snowden (2019). For a detailed look at the Spanish Flu, see Barry (2004).

^2^The Hill, “FBI memo warns QAnon poses potential terror threat: report.”

^3^The New Daily, ‘The coronavirus ‘hoax’: Conspiracy peddlers infecting Australians at alarming rate’.

^4^The Guardian, ‘The QAnon orphans: people who have lost loved ones to conspiracy theories’.

^5^International Business Times, ‘What is The Storm? Conspiracy theory that mysterious White House official QAnon is leaking secrets’.

^6^We focus on Jo Jorgensen in this validation exercise as she is the only minor party candidate to be present on the ballot in all states. Google searches for Jorgensen explain as much as 25% of her vote share. Note that we would not expect this pattern to hold for major parties: for example, in Democrat stronghold California, citizens (even politically active ones) are unlikely to spend much time searching for Democratic Party-related topics on the internet.

^7^Weather.com, ‘Winter Storm Viola Smashed Records in the South and Brought Snow, Ice Into Northeast’.

^8^Where a control variable is missing for a particular observation, we impute the missing control as the average of the non-missing values in the relevant cell. For example, we replace the missing value for Schwartz’s egalitarianism value in Kenya by the average egalitarianism of African countries.

^9^Owing to this continent-wise split, the cluster-robust statistics we use in the remainder of Study 1 are no longer valid, as we now have too few clusters in each regression. We thus calculate 95% CIs using Roodman et al.’s (2019) wild bootstrap procedure, which are cluster-robust even with a small number of clusters.

^10^ https://osf.io/kqnvg

^11^ https://icsmp-covid19.netlify.app/about.html

^12^Data for employment status are missing for Spain. We impute them *via* a multinomial logit regression of employment status for all other countries on the full set of other demographic variables and country fixed effects.

^13^Financial Times, ‘French public feels lied to as lockdown fatigue grows’.
