# Supplementary material for: Early COVID-19 Government Communication Is Associated With Reduced Interest in the QAnon Conspiracy Theory
Source: Front Psychol. 2021 Aug 31;12:681975. doi: 10.3389/fpsyg.2021.681975 (PMC8438198; doi:10.3389/fpsyg.2021.681975)
Supplement: Supplementary file 1 [file Data_Sheet_1.PDF]

Table S1. Summary statistics.

Variable	N	Mean	SD	Min	Max
**Main variables**					
QAnon	16080	2.05	6.31	0	100
COVID-19 is a conspiracy to establish authoritarian gov.	40861	2.74	3.13	0	10
COVID-19 is a hoax for financial gain	40858	2.45	3.07	0	10
Late Campaign (ref.=cases)	16080	-10.52	26.23	-59	59
Late Campaign (ref.=deaths)	15500	-31.11	23.22	-79	20
**Study 2 controls**					
Employment					
Full time	39883	0.44	0.50	0	1
Part time	39883	0.11	0.31	0	1
Unemployed	39883	0.09	0.28	0	1
Student	39883	0.12	0.32	0	1
Retired	39883	0.14	0.35	0	1
Other	39883	0.11	0.31	0	1
Has children	40662	0.54	0.50	0	1
Marital status					
Single	40773	0.34	0.47	0	1
In a relationship	40773	0.20	0.40	0	1
Married	40773	0.46	0.50	0	1
Age	40908	43.11	16.06	18	100
Sex					
Male	40980	0.47	0.50	0	1
Female	40980	0.53	0.50	0	1
Other	40980	0.00	0.05	0	1

Table S1. Summary statistics, continued.

Variable	N	Mean	SD	Min	Max
**Government Restrictions**					
School Closures					
No measures	16033	0.49	0.50	0	1
Recommended closures	16033	0.01	0.09	0	1
Require closing (some)	16033	0.04	0.21	0	1
Require closing (all)	16033	0.46	0.50	0	1
Workplace closing					
No measures	16033	0.53	0.50	0	1
Recommend closing	16033	0.05	0.22	0	1
Require closing (some)	16033	0.20	0.40	0	1
Require closing (all)	16033	0.22	0.41	0	1
Cancel public events					
No measures	16033	0.48	0.50	0	1
Recommend cancelling	16033	0.03	0.18	0	1
Require cancelling	16033	0.49	0.50	0	1
Restrictions on gatherings					

No restrictions	16033	0.54	0.50	0	1
Restrictions on gatherings above 1000 people	16033	0.02	0.13	0	1
Restrictions on gatherings between 101-1000 people	16033	0.03	0.16	0	1
Restrictions on gatherings between 11-100 people	16033	0.12	0.32	0	1
Restrictions on gatherings of 10 people or less	16033	0.30	0.46	0	1
Close public transport					
No measures	16032	0.66	0.47	0	1
Recommend closing	16032	0.16	0.37	0	1
Require closing	16032	0.18	0.38	0	1
Stay at home requirements					
No measures	16033	0.57	0.49	0	1
Recommend not leaving house	16033	0.13	0.33	0	1
Require not leaving (loose)	16033	0.23	0.42	0	1
Require not leaving (strict)	16033	0.07	0.26	0	1

Table S1. Summary statistics, continued.

Variable	N	Mean	SD	Min	Max
Restrictions on internal movement					
No measures	16032	0.58	0.49	0	1
Recommend movement restriction	16032	0.09	0.29	0	1
Restrict movement	16032	0.33	0.47	0	1
International travel controls					
No measures	16033	0.36	0.48	0	1
Screening	16033	0.07	0.25	0	1
Quarantine on high-risk regions	16033	0.06	0.23	0	1
Ban on high-risk regions	16033	0.22	0.41	0	1
Total border closure	16033	0.30	0.46	0	1
**International Country Risk Guide variables**					
Bureaucracy quality	14775	2.60	0.92	1	4
Civil disorder	14775	2.81	0.40	2	4
Civil war	14775	3.69	0.50	3	4
Consumer confidence	14775	2.07	0.19	2	3
Contract viability	14775	3.10	0.56	2	4
Corruption	14775	3.03	1.16	1	6
Cross-border conflict	14775	3.12	0.60	2	4
Economic risk rating	14775	36.36	5.68	16	47
Ethnic tensions	14775	4.06	1.15	1	6
Foreign pressures	14775	2.94	0.39	2	4
Government stability	14775	7.08	0.94	4	10
Military in politics	14775	4.18	1.49	1	6
Payment delays	14775	2.75	0.64	2	4
Religious tensions	14775	4.57	1.29	1	6

**Schwartz values**					
Harmony	9120	-0.01	0.99	-3	2
Embeddedness	9120	-0.14	0.92	-2	2
Hierarchy	9120	-0.01	0.99	-2	3
Mastery	9120	0.10	0.93	-2	3
Affective autonomy	9120	0.15	0.91	-3	2
Intellectual autonomy	9120	0.10	0.95	-2	2
Egalitarianism	9120	0.03	1.03	-2	2

Table S1. Summary statistics, continued.

Variable	N	Mean	SD	Min	Max
**Other controls**					
ln(Gross Domestic Product per capita)	15645	9.74	0.90	7	12
Democracy	15355	5.14	6.20	-10	10
Human Capital Index	14485	2.91	0.54	2	4
ln(COVID-19 cases per capita)	15500	2.46	2.80	0	10

Table S2. Variable definitions and sources.

Variable	Definition	Source
**Main variables**		
QAnon	Google search volumes for the QAnon topic. Measured at the country level. Daily frequency between January 1 and May 24, 2020	Google Trends
COVID-19 is a conspiracy to establish authoritarian gov.	Agreement with the statement: "The coronavirus (COVID-19) is a conspiracy to take away citizen's rights for good and establish an authoritarian government." Scale: 0 - 10; higher values indicate more agreement.	Van Bavel et al. (2020)
COVID-19 is a hoax for financial gain	The coronavirus (COVID-19) is a hoax invented by interest groups for financial gains." Scale: 0 - 10; higher values indicate more agreement.	Van Bavel et al. (2020)
Late Campaign (ref.=cases)	Number of days between the first case of COVID-19 in a country and the first instance of government communication about COVID-19 with the public.	Calculated from Hale et al. (2020)
Late Campaign (ref.=deaths)	Number of days between the first death of COVID-19 in a country and the first instance of government communication about COVID-19 with the public.	Calculated from Hale et al. (2020)
**Study 2 controls**		
Employment	See Table S1	Van Bavel et al. (2020)
Has children	Yes / No	Van Bavel et al. (2020)
Marital status	See Table S1	Van Bavel et al. (2020)
Age		Van Bavel et al. (2020)
Sex	See Table S1	Van Bavel et al. (2020)
Government Restrictions	The OxCGRT dataset collects data on eight types of restrictions: (1) school closures, (2) worplace closures, (3) cancel public events, (4) restrictions on gathering, (5) public transport closures, (6) stay at home requirements, (7) restrictions on internal movement, (8) international travel restrictions. Each restriction is a categorical variable with varying levels of strictness, as shown in Table S1. We use dummy variables for each level of each restriction.	Hale et al. (2020)

Table S2. Variable definitions and sources, continued.

Variable	Definition	Source
**International Country Risk Guide variables**	Expert ratings as detailed below. Definitions from the PRS Group website: https://epub.prsgroup.com/list-of-all-variable-definitions	
Bureaucracy quality	Institutional strength and quality of the bureaucracy is a shock absorber that tends to minimize revisions of policy when governments change. In low-risk countries, the bureaucracy is somewhat autonomous from political pressure.	PRS (2018)
Civil disorder	The potential risk to governance or investment from mass protest, such as anti-government demonstrations, strikes, etc	PRS (2018)
Civil war	The actual or potential risk of civil war (where a rebel force, which holds territory, is in armed conflict with the security forces of the government, and where both forces are citizens of the state in which the conflict occurs).	PRS (2018)
Consumer confidence	The level of consumer confidence vis-à-vis credible surveys, where available, or approximations based on employment trends, economic growth and investment, etc	PRS (2018)
Contract viability	The risk of unilateral contract modification or cancellation and, at worst, outright expropriation of foreign owned assets.	PRS (2018)
Corruption	A measure of corruption within the political system that is a threat to foreign investment by distorting the economic and financial environment, reducing the efficiency of government and business by enabling people to assume positions of power through patronage rather than ability, and introducing inherent instability into the political process.	PRS (2018)
Cross-border conflict	Actual or potential conflict with another nation state that does not affect the whole nation and which can range in severity from cross-border armed conflict and incursion to territorial claims subject to civil mediation or litigation.	PRS (2018)

Table S2. Variable definitions and sources, continued.

Variable	Definition	Source
Economic risk rating	A means of assessing a country's current economic strengths and weaknesses. In general, where strengths outweigh weaknesses, a country will show low risk and where weaknesses outweigh strengths, the economic risk will be high. To ensure comparability between countries, risk components are based on accepted ratios between the measured data within the national economic/financial structure, and then the ratios are compared, not the data. Risk points are assessed for each of the component factors of GDP per head of population, real annual GDP growth, annual inflation rate, budget balance as a percentage of GDP, and current account balance as a percentage of GDP. Risk ratings range from a high of 50 (least risk) to a low of 0 (highest risk), though lowest de facto ratings are generally near 15.	PRS (2018)
Ethnic tensions	A measure of the degree of tension attributable to racial, national, or language divisions. Lower ratings (higher risk) are given to countries where tensions are high because opposing groups are intolerant and unwilling to compromise.	PRS (2018)
Foreign pressures	Actual or potential risk posed by pressures brought to bear on the government by one or more foreign states to force a change of policy. Such pressures can range from diplomatic pressures, through suspension of aid and/or credits, to outright sanctions.	PRS (2018)
Government stability	A measure of both of the government's ability to carry out its declared program(s), and its ability to stay in office.	PRS (2018)
Military in politics	A measure of the military's involvement in politics. Since the military is not elected, involvement, even at a peripheral level, diminishes democratic accountability. Military involvement might stem from an external or internal threat, be symptomatic of underlying difficulties, or be a full-scale military takeover. Over the long term, a system of military government will almost certainly diminish effective governmental functioning, become corrupt, and create an uneasy environment for foreign businesses.	PRS (2018)
Payment delays	The risk associated with receiving and exporting payments from the country (impediments include poor liquidity, exchange controls, an inadequate banking system, etc.)	PRS (2018)
Religious tensions	A measure of religious tensions arising from the domination of society and/or governance by a single religious group -- or a desire to dominate -- in a way that replaces civil law by religious law, excludes other religions from the political/social processes, suppresses religious freedom or expressions of religious identity. The risks involved range	PRS (2018)

from inexperienced people imposing inappropriate policies to civil dissent or civil war.

Table S2. Variable definitions and sources, continued.

Variable	Definition	Source
**Schwartz cultural value orientations**		
Harmony	Individuals are content to accept and fit into the natural and social world	Schwartz (2006)
Embeddedness	People are viewed as entities embedded in the collective.	Schwartz (2006)
Hierarchy	Individuals are socialized to comply with the roles assigned to them in the social hierarchy	Schwartz (2006)
Mastery	Individuals value succeeding and getting ahead through self-assertion	Schwartz (2006)
Affective Autonomy	Individuals pursue affectively positive experience for themselves	Schwartz (2006)
Intellectual Autonomy	Individuals pursue their own ideas and intellectual directions independently.	Schwartz (2006)
Egalitarianism	Individuals are seen as moral equals	Schwartz (2006)
**Other controls**		
ln(Gross Domestic Product per capita)	Measured in real (inflation-adjusted) terms.	Bolt and van Zanden (2014)
Democracy	Level of democracy ranging from -10 (full autocracy) to +10 (full democracy).	Marshall, Gurr and Jaggers (2013)
Human Capital Index	An index based on the average years of schooling and an assumed rate of return to equation. For exact formulae and primary data sources, see	Feenstra, Inklaar and Timmer (2015)

	https://www.rug.nl/ggdc/docs/human_capital_in_pwt_90.pdf	
ln(COVID-19 cases per capita)	Daily frequency	Hale et al. (2020)

Table S3. List of countries and territories and country-level summary statistics.

Country	Studies 1 and 2		Study 1			Study 2						
	Late Campaign		*QAnon*			*Authoritarian*				*Financial*		
	Ref.= first case	Ref.= first death	Mean	S.D.	z-score	N	Mean	S.D.	z-score	Mean	S.D.	z-score
Afghanistan	6	-21	0.12	1.41	-0.43							
Albania	-24	-26	0.15	0.91	-0.42							
Algeria	9	-7	0.09	0.39	-0.43							
Argentina	-40	-45	0.77	1.16	-0.28	635	2.06	2.89	-0.68	1.88	2.84	-0.60
Australia	0	-36	15.48	9.21	2.96	1793	3.22	3.14	0.32	2.24	2.86	-0.31
Austria	-1	-17	22.39	19.07	4.49	1283	1.09	2.26	-1.52	0.92	2.09	-1.38
Azerbaijan	-1	-15	0.07	0.44	-0.44							
Bahrain	-33	-54	0.43	2.28	-0.36							
Bangladesh	-47	-57	0.01	0.11	-0.45	354	2.72	3.07	-0.11	2.20	2.72	-0.34
Barbados	-55	-74	0.94	5.04	-0.24							
Belarus	52	20	0.09	0.48	-0.43							
Belgium	-7	-43	1.95	3.13	-0.02	1074	1.80	2.64	-0.91	1.47	2.44	-0.94
Bolivia	-1	-19	0.19	0.81	-0.41							

Bosnia and Herzegovina	-34	-50	0.85	2.72	-0.26								
Brazil	-28	-48	0.82	0.82	-0.27	1605	1.93	2.88	-0.80	1.50	2.63	-0.91
Bulgaria	-26	-29	0.26	0.94	-0.39	448	2.57	3.21	-0.24	2.87	3.40	0.20
Cambodia	59		0.08	0.55	-0.43							
Canada	45	2	21.58	16.07	4.31	841	2.13	2.79	-0.62	1.60	2.47	-0.83
Chile	11	-8	0.69	1.36	-0.30							
China	4	-6	0.14	1.23	-0.42	960	2.31	2.40	-0.46	2.42	2.20	-0.16
Colombia	-45	-61	0.49	0.77	-0.34	1085	2.12	2.92	-0.63	2.15	3.01	-0.38
Costa Rica	-32	-45	1.40	3.99	-0.14							
Croatia	-29	-52	1.32	2.79	-0.16	423	3.89	3.36	0.90	4.51	3.43	1.53
Cyprus	-4	-17	0.83	3.46	-0.27							
Czechia	-37	-58	2.02	3.64	-0.01							
Denmark	0	-16	0.87	1.87	-0.26	486	1.55	2.20	-1.12	1.38	2.05	-1.01
Dominican Republic	1	-15	0.12	0.72	-0.43							
Ecuador	-35	-48	0.07	0.31	-0.44	118	2.97	3.47	0.11	2.13	3.22	-0.40
Egypt	39	16	0.06	0.22	-0.44							
El Salvador	-56	-68	0.09	0.60	-0.43							
Estonia	14	-13	0.58	2.58	-0.32							
Finland	-2	-54	2.94	3.58	0.20	616	0.87	1.88	-1.71	0.79	1.77	-1.49
France	0	-22	2.32	2.09	0.06	948	3.16	3.19	0.27	2.22	2.88	-0.33
Georgia	-28	-66	0.13	0.71	-0.42							

Germany	-4	-45	15.00	13.05	2.86	1397	1.91	2.98	-0.81	1.66	2.79	-0.78
Ghana	-2	-10	0.25	1.34	-0.40							
Greece	-1	-15	2.19	3.53	0.03	609	2.09	2.65	-0.65	1.94	2.62	-0.55
Guam	-38	-44	0.53	3.65	-0.34							
Guatemala	-46	-48	0.12	0.55	-0.42							
Honduras	-9	-24	0.06	0.40	-0.44							
Hong Kong	-22	-34	0.57	1.62	-0.33							
Hungary	-5	-16	0.43	1.02	-0.36	437	3.43	3.37	0.50	2.66	3.15	0.03
Iceland	-36	-52	0.58	2.63	-0.32							
India	-5	-46	0.37	0.42	-0.37	564	3.88	2.98	0.90	3.14	2.85	0.42
Indonesia	-59	-68	0.13	0.26	-0.42							
Iran	7	7	0.05	0.21	-0.44							
Iraq	-5	-14	0.10	0.41	-0.43	454	5.00	3.50	1.86	5.23	3.55	2.12
Ireland	-25	-36	8.02	8.69	1.32	685	1.36	2.42	-1.28	1.06	2.19	-1.27
Israel	-25	-54	1.72	2.82	-0.07	1159	2.16	2.59	-0.59	1.80	2.55	-0.67
Italy	0	-21	3.52	3.56	0.33	1049	2.53	3.01	-0.28	2.46	2.93	-0.13
Jamaica	-35	-43	0.40	2.37	-0.36							
Japan	20	-9	3.48	3.63	0.32	1000	3.99	2.62	0.99	2.58	2.34	-0.03
Jordan	2	-22	0.40	1.67	-0.36							
Kazakhstan	3	-4	0.37	1.19	-0.37							
Kenya	-17	-30	0.16	0.66	-0.42							

Kuwait	-32	-72	0.44	1.64	-0.35							
Latvia	9	-23	0.48	2.01	-0.35	844	3.18	3.27	0.29	3.52	3.36	0.73
Lebanon	0	-18	0.19	0.92	-0.41							
Lithuania	-2	-24	3.47	6.88	0.31							
Luxembourg	5	-9	1.18	4.25	-0.19							
Malaysia	-9	-61	0.19	0.43	-0.41							
Mauritius	8	5	0.05	0.58	-0.44							
Mexico	25	5	1.01	1.10	-0.23	1094	2.06	2.92	-0.68	1.98	2.94	-0.52
Moldova	-44	-54	0.07	0.51	-0.44							
Morocco	2	-6	0.11	0.53	-0.43	556	3.60	3.11	0.65	3.93	3.21	1.07
Myanmar (Burma)	-15	-22	0.32	1.93	-0.38							
Namibia	3		0.29	2.12	-0.39							
Nepal	-9	-121				314	3.18	3.09	0.28	3.41	3.21	0.64
Netherlands	11	3	6.73	5.75	1.03	1197	1.03	2.04	-1.57	0.82	1.87	-1.47
New Zealand	-37	-67	4.96	6.51	0.64	448	2.72	3.07	-0.11	2.14	2.84	-0.39
Nigeria	-37	-61	0.18	0.79	-0.41	489	4.64	3.29	1.55	4.77	3.34	1.74
Norway	-26	-42	1.82	3.32	-0.05	479	0.85	1.80	-1.73	0.90	1.96	-1.40
Oman	-35	-71	0.15	0.89	-0.42							
Pakistan	-14	-36	0.90	2.02	-0.25	420	4.02	3.05	1.02	4.32	3.26	1.38
Panama	-49	-50	0.20	1.14	-0.41							
Paraguay	-45	-58	1.01	3.02	-0.23							

Peru	-1	-15	0.69	1.32	-0.30									
Philippines	-6	-9	0.61	0.95	-0.32	445	5.11	3.14	1.96	4.44	3.36	1.48
Poland	-41	-49	3.27	4.37	0.27	1497	4.69	3.29	1.59	4.33	3.30	1.39
Portugal	-36	-51	1.08	2.22	-0.21							
Puerto Rico	-14	-26	2.06	6.11	0.00							
Qatar	-30	-58	3.55	6.70	0.33							
Romania	-1	-26	2.32	3.10	0.06	852	4.61	3.54	1.52	4.95	3.59	1.90
Russia	45	-3	0.29	0.62	-0.39	470	2.84	3.17	-0.01	2.86	3.23	0.19
Saudi Arabia	-32	-54	0.10	0.33	-0.43							
Senegal	12	-18				267	2.68	2.89	-0.15	3.15	3.25	0.43
Serbia	-10	-24	4.49	7.06	0.54	621	3.21	3.20	0.31	3.43	3.26	0.66
Singapore	-21	-79	0.80	1.46	-0.28	444	3.26	2.86	0.36	2.77	2.71	0.13
Slovakia	-39	-51	0.85	2.16	-0.26	985	4.23	3.43	1.19	3.49	3.37	0.71
Slovenia	-1	-10	2.41	4.84	0.08							
South Africa	0	-22	4.50	4.64	0.54	421	2.18	2.88	-0.58	1.72	2.63	-0.73
South Korea	12	-19	0.40	0.92	-0.36	403	3.00	2.66	0.13	2.80	2.56	0.15
Spain	-1	-32	2.03	2.28	0.00	1019	1.60	2.50	-1.08	1.21	2.18	-1.14
Sri Lanka	1	-60	0.01	0.17	-0.45							
Sweden	38	-2	3.94	3.61	0.42	1473	1.16	2.25	-1.46	0.77	1.90	-1.50
Switzerland	2	-7	13.93	12.61	2.62	935	2.47	3.03	-0.33	2.03	2.90	-0.48
Taiwan	-20	-45	0.06	0.21	-0.44	685	4.88	3.23	1.76	3.74	3.14	0.91

Tanzania	10	-3	0.03	0.33	-0.45							
Thailand	53	5	0.19	0.38	-0.41							
Trinidad and Tobago	-40	-52	0.18	1.51	-0.41							
Tunisia	1	-15	0.01	0.17	-0.45							
Turkey	-33	-39	0.10	0.22	-0.43	1244	4.26	3.28	1.23	4.46	3.34	1.50
Uganda	-52		0.21	1.46	-0.41							
Ukraine	0	-10	0.42	0.95	-0.36	504	4.23	3.32	1.19	4.64	3.39	1.65
United Arab Emirates	44	-9	0.29	1.01	-0.39							
United Kingdom	2	-32	8.29	5.92	1.38	519	1.53	2.27	-1.14	1.04	1.95	-1.29
United States	55	16	26.62	19.23	5.42	1125	3.54	3.31	0.60	2.83	3.19	0.17
Uruguay	0	-15	0.16	0.95	-0.42							
Uzbekistan	-54	-66	0.02	0.25	-0.45							
Venezuela	-15	-28	0.45	1.36	-0.35							
Vietnam	6		0.15	0.32	-0.42							
Zambia	-26	-41	0.05	0.58	-0.44							
Zimbabwe	-53	-56	0.19	1.34	-0.41							

Table S4. Results exactly following the pre-registration plan.

	A. *Late Campaign* relative to first COVID-19 case in country			
	(1)	(2)	(3)	(4)
	Authoritarian	Financial	Authoritarian	Financial
Late Campaign	-0.0114	-0.0141*	-0.0104	-0.0124*
	[0.0077]	[0.0071]	[0.0073]	[0.0067]
Demographic controls			Yes	Yes
Observations	37,140	37,140	35,586	35,587
R2	0.0078	0.0125	0.0263	0.0339

	B. *Late Campaign* relative to first COVID-19 death in country			
	(1)	(2)	(3)	(4)
	Authoritarian	Financial	Authoritarian	Financial
Late Campaign	-0.0074	-0.0081	-0.0067	-0.0070
	[0.0070]	[0.0066]	[0.0067]	[0.0062]
Demographic controls			Yes	Yes
Observations	37,140	37,140	35,586	35,587
R2	0.0029	0.0037	0.0222	0.0269

Notes. Demographic controls: age (continuous), gender (categorical), has children (binary), employment status (categorical). All specifications include a constant term.

Table S5. Results for the sample of 49 countries which appear in both Study 1 and Study 2.

A. *Late Campaign* relative to first COVID-19 case in country

	(1) Authoritarian	(2) Financial	(3) Authoritarian	(4) Financial	(5) QAnon	(6) QAnon
Late Campaign	-0.0110	-0.0138*	0.0048	0.0135**	0.0210***	0.0210**
	[0.0080]	[0.0074]	[0.0055]	[0.0053]	[0.0071]	[0.0083]
Observations	39,069	39,066	38,528	38,525	7,041	7,041
R-squared	0.0073	0.0121	0.1361	0.1524	0.720	0.751
N. Countries	49	49	49	49	49	49
Continent FE			Yes	Yes		Yes
Country Controls			Yes	Yes		Yes
Demographics			Yes	Yes		
Day FE						Yes

B. *Late Campaign* relative to first COVID-19 death in country

	(1) Authoritarian	(2) Financial	(3) Authoritarian	(4) Financial	(5) QAnon	(6) QAnon
Late Campaign	-0.0075	-0.0076	-0.0019	0.0088	0.0161**	0.0167**
	[0.0085]	[0.0079]	[0.0060]	[0.0058]	[0.0070]	[0.0080]
Observations	39,069	39,066	38,528	38,525	7,041	7,041
R-squared	0.0026	0.0028	0.1360	0.1514	0.719	0.750
N. Countries	49	49	49	49	49	49
Continent FE			Yes	Yes		Yes
Country Controls			Yes	Yes		Yes
Demographics			Yes	Yes		
Day FE						Yes

References

Bolt, J., & Van Zanden, J. L. (2014). The Maddison Project: collaborative research on historical national accounts. *The Economic History Review*, *67*(3), 627-651.

Feenstra, R. C., Inklaar, R., & Timmer, M. P. (2015). The next generation of the Penn World Table. *American Economic Review*, *105*(10), 3150-82.

Hale, T., Webster, S., Petherick, A., Phillips, T., & Kira, B. (2020). Oxford COVID-19 Government Response Tracker, Blavatnik School of Government. Data use policy: Creative Commons Attribution CC BY standard.

Marshall, M. G., Gurr, T. R., & Jaggers, K. (2013). Polity IV project: Political regime characteristics and transitions, 1800–2012. *Center for Systemic Peace*.

PRS (2018). International Country Risk Guide: Guide to Data Variables. Available at: https://epub.prsgroup.com/list-of-all-variable-definitions.

Schwartz, S. (2006). A theory of cultural value orientations: Explication and applications. *Comparative Sociology*, *5*(2-3), 137-182.

Van Bavel, J.J., Cichocka, A., Capraro, V., Sjåstad, H., Nezlek, J.B., Pavlović T., Alfano A., Gelfand, M.J., Azevedo, F., Birtel M.D., Cislak, A., Lockwood, P., Ross, R. M., Stoyanova, K.K., Abts, K., Amodio, D.M., Apps, M. A.J., Aruta, J.J.B.R., Besharati, S., ..., Boggio, P. (2020). National identity predicts public health support during a global pandemic. Manuscript submitted for publication.
